# Estimating Above-Ground Carbon Biomass in a Newly Restored Coastal Plain Wetland Using Remote Sensing

**DOI:** 10.1371/journal.pone.0068251

**Published:** 2013-06-28

**Authors:** Joseph B. Riegel, Emily Bernhardt, Jennifer Swenson

**Affiliations:** 1 Nicholas School of the Environment, Duke University, Durham, North Carolina, United States of America; 2 Department of Biology, Duke University, Durham, North Carolina, United States of America; University of Florida, United States of America

## Abstract

Developing accurate but inexpensive methods for estimating above-ground carbon biomass is an important technical challenge that must be overcome before a carbon offset market can be successfully implemented in the United States. Previous studies have shown that LiDAR (light detection and ranging) is well-suited for modeling above-ground biomass in mature forests; however, there has been little previous research on the ability of LiDAR to model above-ground biomass in areas with young, aggrading vegetation. This study compared the abilities of discrete-return LiDAR and high resolution optical imagery to model above-ground carbon biomass at a young restored forested wetland site in eastern North Carolina. We found that the optical imagery model explained more of the observed variation in carbon biomass than the LiDAR model (adj-R^2^ values of 0.34 and 0.18 respectively; root mean squared errors of 0.14 Mg C/ha and 0.17 Mg C/ha respectively). Optical imagery was also better able to predict high and low biomass extremes than the LiDAR model. Combining both the optical and LiDAR improved upon the optical model but only marginally (adj-R^2^ of 0.37). These results suggest that the ability of discrete-return LiDAR to model above-ground biomass may be rather limited in areas with young, small trees and that high spatial resolution optical imagery may be the better tool in such areas.

## Introduction

The destruction of forests and wetlands is a primary contributor to global climate change [1], [2], [3]. For this reason, national and international climate change mitigation plans often include as key components programs designed to protect and restore these ecosystems. The United Nation’s REDD program as well as recent proposals in the United States for a carbon offset market are examples of such programs. Broadly speaking, they aim to promote conservation by placing a monetary value on the carbon sequestration services provided by healthy ecosystems [4], [5], [6].

Due to the significant monetary and ecological values involved, successfully implementing these programs requires accurate and, ideally, inexpensive methods for estimating the amount of carbon sequestered in plant biomass [7], [8]. Historically, estimating carbon biomass over large areas involved collecting biomass density data in the field and then applying expansion factors to scale up. More recently, methods have been developed for estimating carbon biomass using remotely sensed data. This approach involves creating empirical models that relate variables extracted from the remotely sensed data to sample biomass data. The models can then be used to produce spatially explicit maps of carbon biomass, from which estimates of total biomass can be derived. Compared with earlier methods, the use of remotely sensed data has the potential for producing more accurate estimates of total biomass; however, remote sensing methods can also be more expensive due to the cost of acquiring and processing the remotely sensed data.

Light detection and ranging (LiDAR) is a relatively new remote sensing technology that is widely regarded to be well-suited for estimating biomass over large areas [9], [10]. Airborne LiDAR instruments operate by directing pulses of laser light toward the ground and then recording the amount of time required for the pulses to strike objects on the ground and then reflect back to a sensor. Using this return time along with information from the aircraft’s navigational equipment, it is possible to calculate the three-dimensional coordinates of those objects on the ground [11], [12]. Airborne LiDAR instruments can be divided into two types based on the characteristics of the emitted laser pulses and the amount of information they record from the returning electromagnetic waves. Full-waveform LiDAR instruments record the entire electromagnetic wave that returns to the sensor, and their emitted laser beams are typically spread over a larger area (on the order of tens of meters) when they reach the ground. By contrast, discrete-return LiDAR instruments record only the individual peaks in the returning wave, and they usually have a much smaller footprint (usually <1 m) [12]. While full-waveform LiDAR is used mainly as a research tool for studying vegetation patterns, discrete-return LiDAR technology has become widely available for a variety of commercial and research purposes.

LiDAR-based biomass models have generally been quite successful, with R^2^ values of greater than 0.8 reported for tall-stature mature forests [10], [13], [14], [15]. LiDAR-based biomass models have also generally out-performed models based on other remote sensing technologies, such as radar or optical imagery [10], [16], [17]. LiDAR technology is unique in that its pulses can penetrate tree canopies and retrieve highly accurate and detailed information about a forest’s vertical structure in addition to its horizontal structure [12], [18], [19]. In mature forests with mid to high biomass densities (e.g. 100 Mg/ha), vertical structural characteristics are highly correlated with total biomass, and so LiDAR has the potential for producing more accurate estimates of biomass than remote sensing technologies that can only retrieve information about a forest’s horizontal structure.

Many early successful LiDAR biomass studies were conducted using full-waveform instruments over areas with mature forests [10], [13], [14], [15]. More recently, researchers have shown that discrete-return LiDAR can also reliably estimate biomass and measure forest canopy characteristics from which biomass can be estimated [20], [21], [22], [23]. While these studies have succeeded in showing that LiDAR is a valuable tool for modeling biomass in mature forests, the abilities of either type of LiDAR to estimate biomass in areas with relatively short-stature vegetation have not yet been adequately evaluated. While there have been several studies that employed discrete-return LiDAR in areas with relatively sparse vegetation or small (<6 m) trees [24], [25], [26], [27], these studies focused on modeling forest characteristics (e.g, heights) other than biomass. In the context of a carbon offset market, being able to produce reliable estimates of carbon biomass in areas with relatively small trees, such as areas that have been recently reforested or afforested, would be essential.

The main goal of this current study was to evaluate the ability of discrete-return LiDAR to estimate the amount of carbon sequestered over a four-year period at a recently restored forested wetland. In doing this, we had two specific objectives. First, we aimed to determine how successfully, relative to previous LiDAR studies of mature forests, discrete-return LiDAR could model above-ground carbon biomass at a study site with relatively young, small trees. Because, other things being equal, smaller objects are more difficult to detect using LiDAR or any other remote sensing technology, our hypothesis was that a LiDAR-based model would not perform as well in areas with smaller trees. Second, we aimed to determine whether LiDAR could model above-ground carbon biomass at the study site better than high-resolution optical imagery. Because the forest at the study site had not yet achieved canopy closure, we hypothesized that the LiDAR model would perform only slightly better, if at all, than the optical imagery model.

## Materials and Methods

### Study Area

The Timberlake Restoration Project is a privately-owned wetland mitigation area located on the Albemarle Peninsula in eastern North Carolina. This region was once covered by pocosin (evergreen shrub-scrub) wetlands; however, a large percentage of those wetlands were extensively logged in the early 1900s and then drained for agriculture in the 1970s and 1980s [28], [29]. Timberlake includes an old agriculture field, approximately 440 ha in size, which is currently being restored to its pre-agricultural forested wetland state. This process began in 2004 after the last corn harvest, and it involved filling in drainage ditches, removing pumps, filling in sections of the main canal, and delineating a zone of preferential water flow [30]. The old agriculture field is mostly flat, with a range of elevation between -0.5 m and 2 m above sea level. Lower elevations are often inundated with water for much of the year [31].

In 2004, approximately 750,000 live saplings were planted at the site. Trees were planted as live stakes approximately eight feet apart. A total of fourteen tree species were planted, which were grouped into three “mixes” at the time of planting: riverine, non-riverine, and cedar. The riverine mix was planted in lower elevation areas, where soil water levels were expected to be higher, while the non-riverine mix was planted in higher elevations. The riverine mix included: *Salix nigra*, *Taxodium distichum*, *Baccharis halimifolia*, *Fraxinus pennsylvania*, *Nyssa aquatic*, *N sylvatica* var. *biflora*, *Persea borbonia*, and *Rhus copallinum*. The non-riverine mix included: *Liquidambar styraciflua*, *Quercus michauxii*, *Q. phellos*, *Q. nigra*, and *Q. falcata*. In addition, two smaller areas at the site were planted with a single species, Atlantic white-cedar (*Chamaecyparis thyoides*).

### Sample Biomass Data Collection

**Figure 1:**
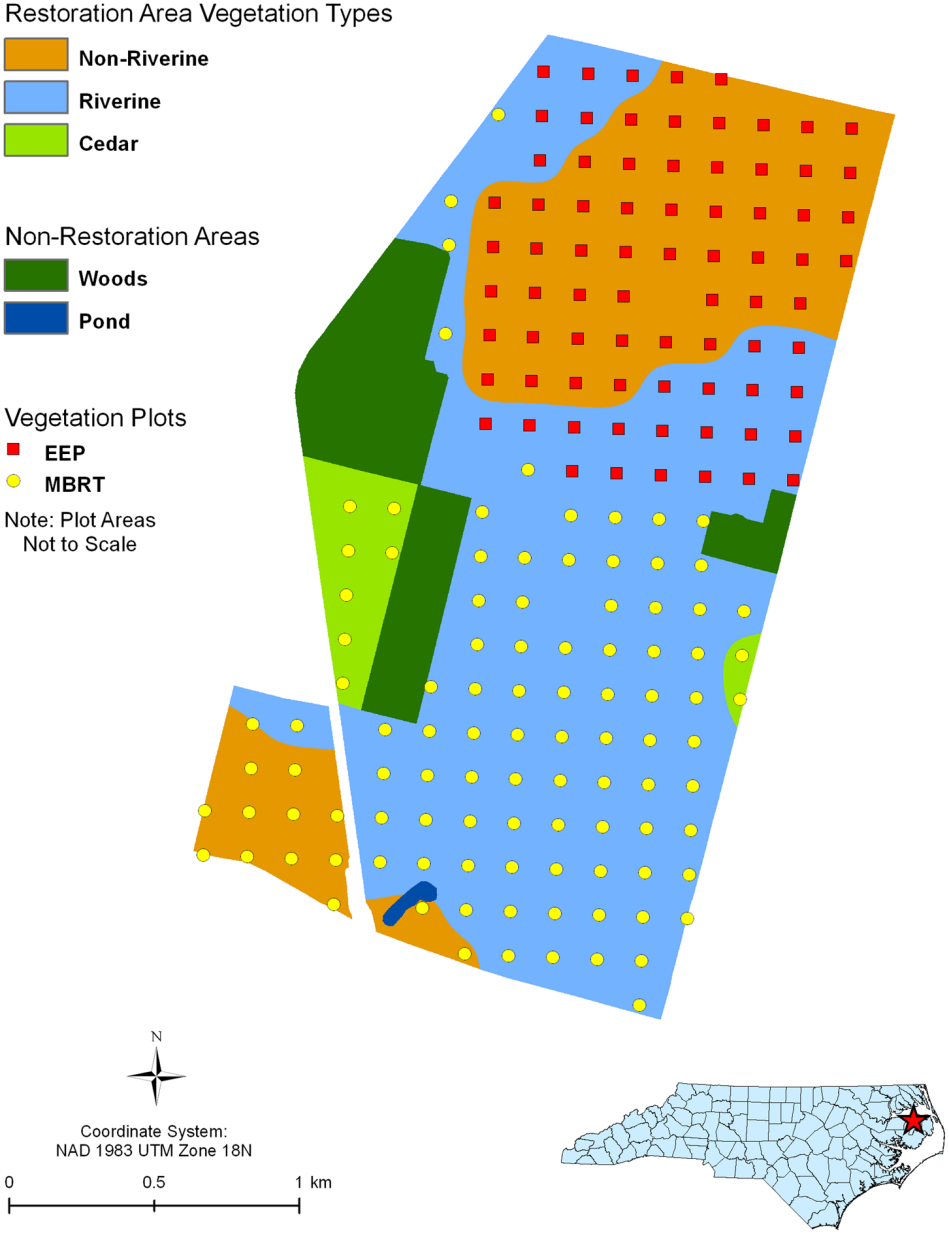
Map of plot locations and dominant vegetation types. Vegetation measurements were collected annually from 10×10 m plots within a section of the wetland mitigation area sold to the NC Ecosystem Enhancement Program (NCEEP), a state in lieu fee trading program (red boxes). These NC EEP plots covered the northern part of the study area and included both riverine and non-riverine areas; however, there were no plots within the portions of the site planted with white cedar.

To comply with wetland mitigation permitting requirements, vegetation monitoring has been conducted at the site annually since 2004 (Figure 1). The northern part of the agricultural field is being sold through the North Carolina Ecosystem Enhancement Program (EEP), which requires vegetation monitoring in square, 10×10 m plots, of which 76 were distributed in a regular east-west grid. For each tree in these plots, the species, height, and diameter at ground level have been recorded annually by an independent contractor. Because the planted trees were initially quite small (<1 m), diameter at ground level was measured instead of diameter at breast height (1.37 m). Tree height was measured using a height pole, and diameter at ground level was measured using a ruler or calipers. In 2008, the average tree height was observed to be 1.5 m, with a maximum height of 4.4 m. Per plot, the number of individual trees measured ranged from 0 to 25. Vegetation monitoring for a particular year was usually conducted in the spring, before the next growing season had begun. Vegetation monitoring in the southern part of the property was conducted under the direction of North Carolina’s Mitigation Bank Review Team (MBRT). These 112 plots differed substantially in area and types of measurements, and were used in this study only to identify riverine and non-riverine areas in the southern part of the study area by their species composition. The study area was characterized as riverine, non-riverine, or cedar using information about which tree species were planted on each EEP and MBRT plot. Each plot was scored and a spline interpolation algorithm was used to map dominant vegetation types for the whole study area (Figure 1). Riverine areas constituted approximately 268 ha (61% of the study area), non-riverine areas approximately 148 ha (34%), and cedar areas approximately 22 ha (5%) (Table 1). All further discussion of field plots refer to the 76 EEP plots in the northern half of the study area.

**Table 1:** Characteristics of restoration area by dominant vegetation type.

Vegetation Type	Area (ha)	Num EEP Plots
Riverine	268	29
Non-Riverine	148	47
Cedar	22	0
Total	438	76

The total above-ground carbon biomass (AGCB) for each plot in 2008 was estimated in three steps. First, species-specific regression equations were developed to estimate each tree’s diameter at breast height (DBH) from its field recorded diameter at ground level. Permission to access the site was provided by the owner of the land (the Great Dismal Mitigation Bank, Limited Liability Company). In the summer of 2011, an average of 20 field measurements of both DBH and diameter at ground level were collected for each tree species. Any stem with a diameter greater than 1.0 cm was recorded. Because several tree species at the site have a growth form of multiple stems (e.g. *Salix nigra*), the cross-sectional area for each stem was calculated from the diameter measurements, and these values were then added together to get total cross-sectional area. Regression equations were then developed to predict total stem area at breast height from total stem area at ground level (Table 2).

**Table 2:** Allometric equations for calculating cross-sectional area at breast-height from cross-sectional area at ground level.

Species Name	b_0_	b_1_	Relationship	n	R^2^
*Baccharis halimifolia*	−537.98	0.5313	Linear*	14	0.47
*Fraxinus pennsylvanica*	−375.53	0.3867	Linear	27	0.90
*Persea borbonia*	−106.66	0.2735	Linear	17	0.94
*Pinus taeda*	−341.1	0.4568	Linear	12	0.82
*Quercus falcata*	−107.28	0.3118	Linear	20	0.69
*Quercus michauxii*	−184.97	0.3831	Linear	19	0.94
*Quercus nigra*	−500	0.4267	Linear	24	0.88
*Quercus phellos*	−89.443	0.3705	Linear	19	0.84
*Rhus copallinum*	−176.75	0.7846	Linear	8	0.74
*Salix nigra*	9.176	0.6963	Power**	17	0.85
*Taxodium distichum*	−273.34	0.2143	Linear	29	0.70

* Equation of the form y = b_1_x+b_0_.

** Equation of the form y = b_0_*x^b1^.

The second step used each tree’s estimated DBH to estimate its above-ground biomass. This was done using published dry-weight biomass allometric equations for each species, if available. For species with no published allometric equations, dry-weight biomass was estimated using the most appropriate generic equations available (Table 3). Estimates of above-ground carbon were calculated for each tree by multiplying the above-ground biomass estimates by 0.5 [32].

**Table 3:** Biomass allometric equations used for each species.

Species Name	Equation Used
*Baccharis halimifolia*	“Mixed Hardwood” [36]
*Fraxinus pennsylvanica*	*Fraxinus pennsylvanica* [37]
*Liquidambar styraciflua*	*Liquidambar styraciflua* [37]
*Nyssa aquatica*	*Nyssa aquatica* [37]
*Nyssa sylvatica* var. *biflora*	*Nyssa aquatica* [37]
*Persea borbonia*	“Mixed Hardwood” [36]
*Pinus taeda*	*Pinus taeda* [38]
*Quercus michauxii*	“Hard Maple, Oak, Hickory, Beach” [36]
*Quercus falcata*	*Quercus falcata* [39]
*Quercus nigra*	*Quercus nigra* [37]
*Quercus phellos*	“Hard Maple, Oak, Hickory, Beach” [36]
*Rhus copallinum*	“Mixed Hardwood” [36]
*Salix nigra*	*Salix spp*. [40]
*Taxodium distichum*	“Cedar, Larch” [36]

The final step in estimating plot level AGCB was to sum the AGCB estimates for all the trees in each respective plot. As of the summer of 2011, much of the study area was covered with dense grasses and sedges in addition to trees. Given the fast pace of change at the restoration site, we decided that it would be too difficult to estimate the amount of herbaceous biomass present in 2008. Thus, for the purpose of this analysis, we only estimated above-ground carbon biomass in woody vegetation for each of the plots (Table 4).

**Table 4:** Descriptive statistics for sample carbon biomass data.

	Riverine	Non-Riverine	All
n	29	47	76
Mean (Mg C/ha)	1.34	0.51	0.83
Std. Dev. (Mg C/ha)	1.91	0.68	1.35
Min (Mg C/ha)	0.03	0.00	0.00
1st Q. (Mg C/ha)	0.19	0.05	0.10
Median (Mg C/ha)	0.58	0.27	0.39
3rd Q. (Mg C/ha)	1.63	0.71	0.91
Max (Mg C/ha)	8.40	2.87	8.40

### Remote Sensing Datasets

Discrete-return LiDAR data was collected by an independent contractor over the study site on November 18, 2008. The data were originally collected to analyze the hydrology of the study area and not for estimating biomass. An Optech GEMINI sensor was mounted on a twin-engine Cessna Skymaster, which flew at an average altitude of 650 m and at an average speed of 59.2 m/s. The pulse and scan frequencies were 100 kHz and 45 Hz respectively, and up to four returns were collected per pulse (NCALM, 2008). Vertical and horizontal point coordinates were estimated to be accurate within approximately 5–10 cm (NCALM, 2008). The LiDAR dataset as a whole had an average pulse density of 5–6 pulses/m^2^ and approximately 10 total returns/m^2^. The average footprint diameter was calculated to be approximately 16.25 cm [33].

Optical imagery for the study area was acquired from the USDA’s National Agricultural Imagery Program. The imagery had a cell size of 1 m and included four bands: red, green, blue, and near-infrared (NIR). Though the optical imagery was collected after the 2009 growing season had begun, alternative imagery sources were less preferable due to the lack of a near-infrared band or to a spatial resolution that was too coarse (10 m to 30 m).

### Remote Sensing Data Extraction

GPS coordinates were collected at the southwest corner of each of the 76 vegetation plots using a Garmin 272 GPS unit. These GPS coordinates were estimated to be within 2–3 m of the actual corners of the plots. The GPS coordinates were used to create 10×10 meter analysis windows representing the vegetation plots.

The LiDAR data points were separated into ground and vegetation points [34]. The LiDAR points falling within the analysis windows were then isolated and analyzed. The following variables were extracted from the LiDAR data for each plot: percentage of points classified as vegetation points; the mean, maximum, standard deviation, 50^th^ percentile, 75^th^ percentile, and 90^th^ percentile of the LiDAR point intensity values; and the mean, maximum, standard deviation, 50^th^ percentile, 75^th^ percentile, and 90^th^ percentile of the LiDAR point height-above-ground values.

Using the 2009 NAIP imagery, a map of the Normalized Difference Vegetation Index (NDVI) [35] was created for the entire restoration area. The NDVI equation is as follows:
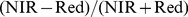
(1)


Healthy green vegetation is unique in that it tends to reflect light in the near-infrared range and absorb light in the red part of the electromagnetic spectrum. For this reason, the NDVI can be used to distinguish healthy green vegetation from other land covers. For each of the 76 EEP plots, the minimum, maximum, mean, and standard deviation NDVI values were calculated.

### Biomass Model Development

Using the statistical software program R, ordinary least squares multiple linear regression models were created that related plot AGCB data to the vegetation variables derived from the remote sensing data. A total of three models were developed: one based on the LiDAR point variables, another based on the NDVI variables extracted from the optical imagery, and lastly, a model including both the LiDAR point variables and the NDVI variables.

In creating the statistical models, the explanatory variables most highly correlated with AGCB were initially included. Variables were then added if doing so increased the adjusted R^2^ value of the resulting model. Many of the LiDAR-derived height variables were highly correlated with each other and adding them only decreased the adjusted R^2^ value. Some variables, such as the LiDAR intensity values, were excluded because they were discovered to be unreliable. The range of intensity values for the field plots was much smaller than the range of values for the entire study area. Using them to estimate total biomass would have required extrapolation well beyond the range of values in the sample data, leading to unreliable estimates of total biomass. In creating the regression models, log and square root transformations of the explanatory and response variables were considered.

### Total Biomass Estimation

An analysis grid consisting of 10 m by 10 m cells was overlain on the entire study area (n = 44763) and for each cell, the remote sensing variables were calculated and the regression equations were used to estimate above-ground carbon biomass. For each regression model, an AGCB estimate for the whole restoration area was calculated by summing the estimates of all the cells. Some of the cells had areas of less than 100 m^2^ because the borders of the restoration area cut through them. An incomplete cell was removed from analysis if the total number of LiDAR points falling within its boundary was less than 50 or if the cell was not large enough to cover 10 1-m^2^ NDVI pixels. For the incomplete cells with sufficient data, the biomass estimates was scaled down based on the proportion of the 100 m^2^ area included in the truncated cell.

## Results

### Sample Biomass Data

**Figure 2:**
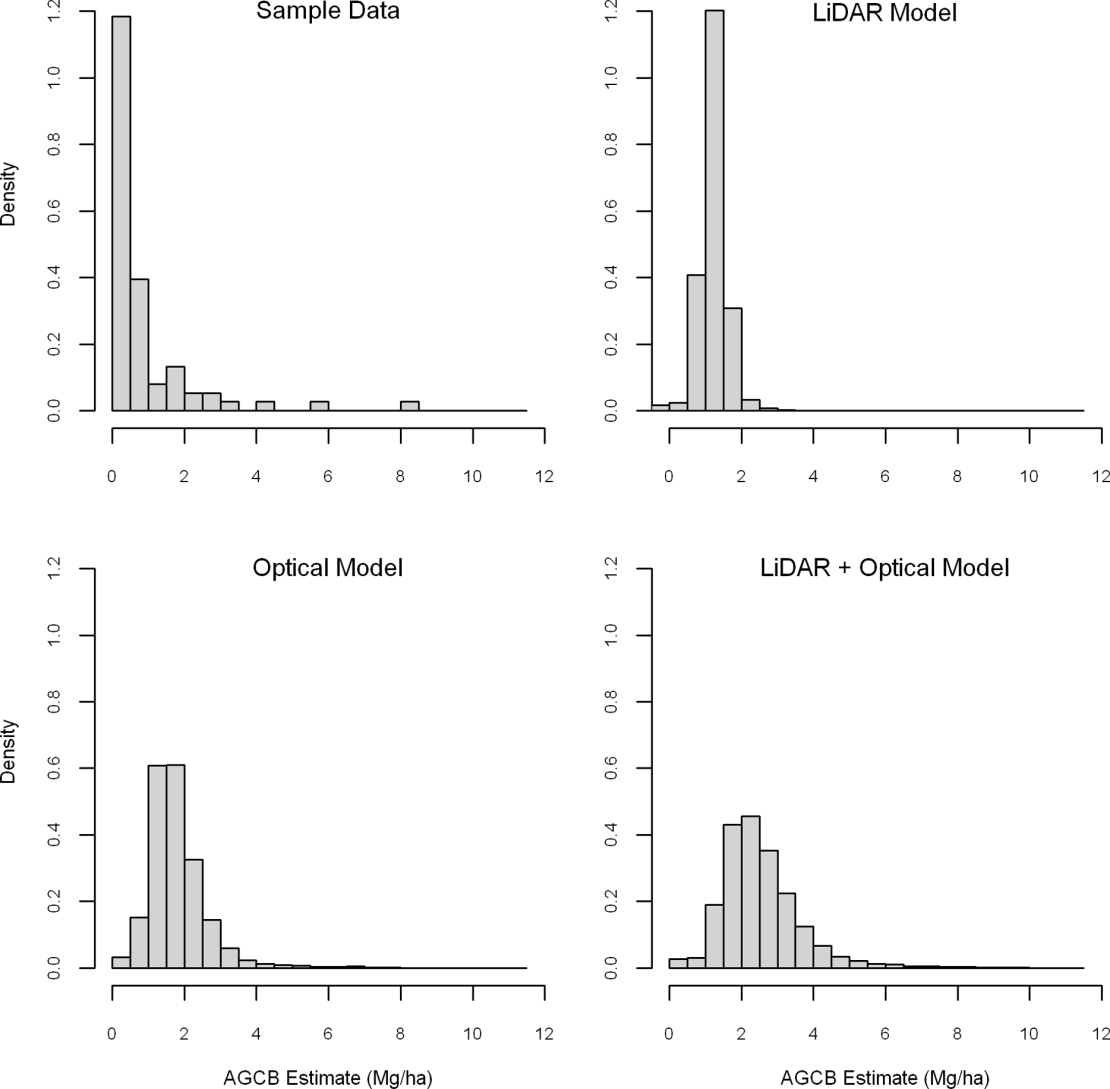
Histograms of sample AGCB data and modeled AGCB estimates. The top left panel is a histogram of the sample above-ground carbon biomass data. The other panels are histograms of the plot biomass data predicted by the three models. Each of the three models had difficulty correctly capturing the observed distribution of AGCB at the study site.

The mean carbon AGCB density over the 76 plots was 0.83 Mg C/ha (Table 4), with individual plots ranging from 0 to 8.4 Mg C/ha. Plots dominated by black willow (*Salix nigra*) trees tended to have the highest carbon biomass. The 29 riverine plots had a mean AGCB 0.83 Mg C/ha greater than the mean AGCB for the non-riverine plots (n = 47, p<0.05). The overall distribution for all field plots was skewed to lower biomass values (Figure 2, upper left panel).

### Remote Sensing Models and Carbon Estimates

**Figure 3:**
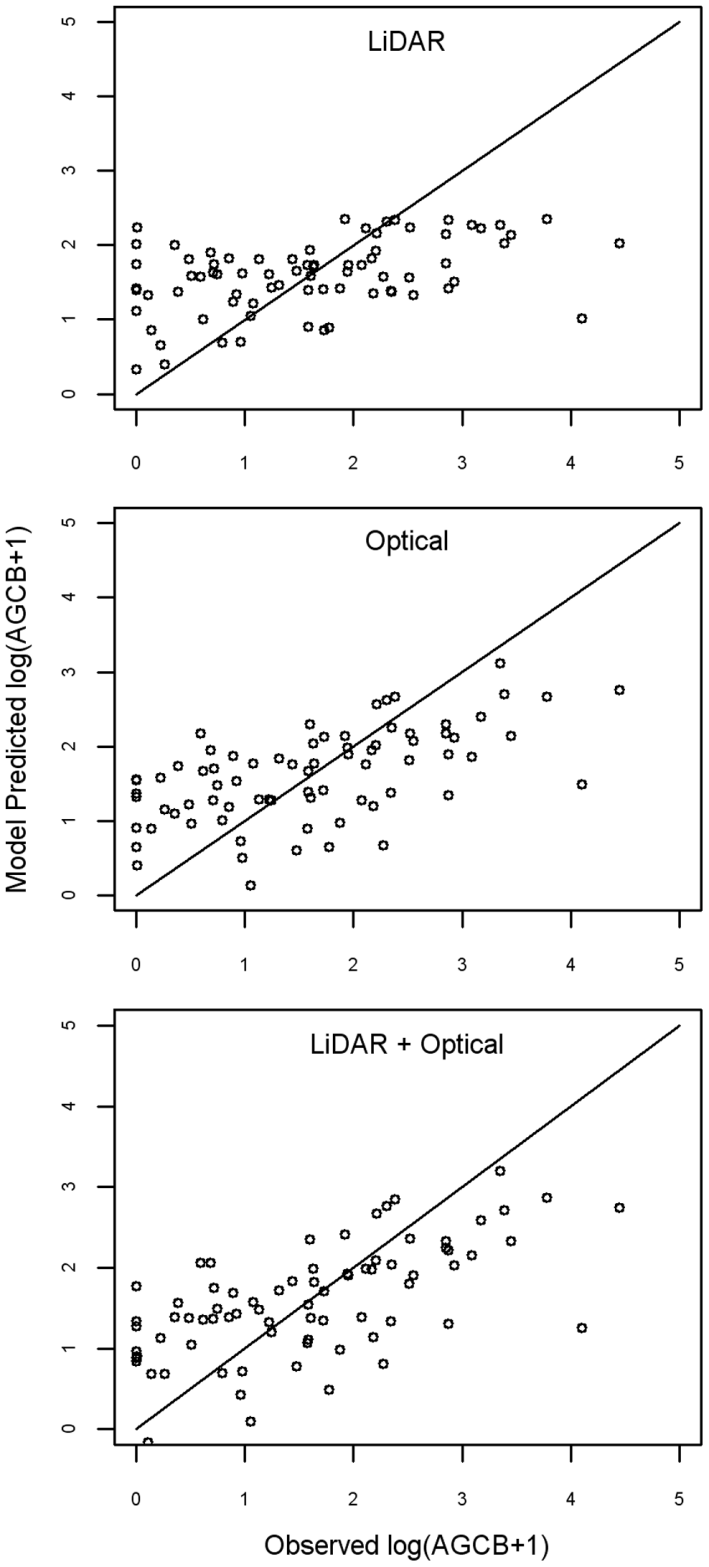
Graphs of predicted vs. observed AGCB for the three remote sensing models. For the three models, the predicted above-ground carbon biomass values for the 76 EEP plots are graphed against the above-ground carbon biomass values estimated using field techniques. Points above the one-to-one line represent plots for which the model over-estimated AGCB. Points below the one-to-one line represent plot for which the model under-estimated AGCB. To varying degrees, all the models over-estimated low observed AGCB values and under-estimated high observed AGCB values.

**Figure 4:**
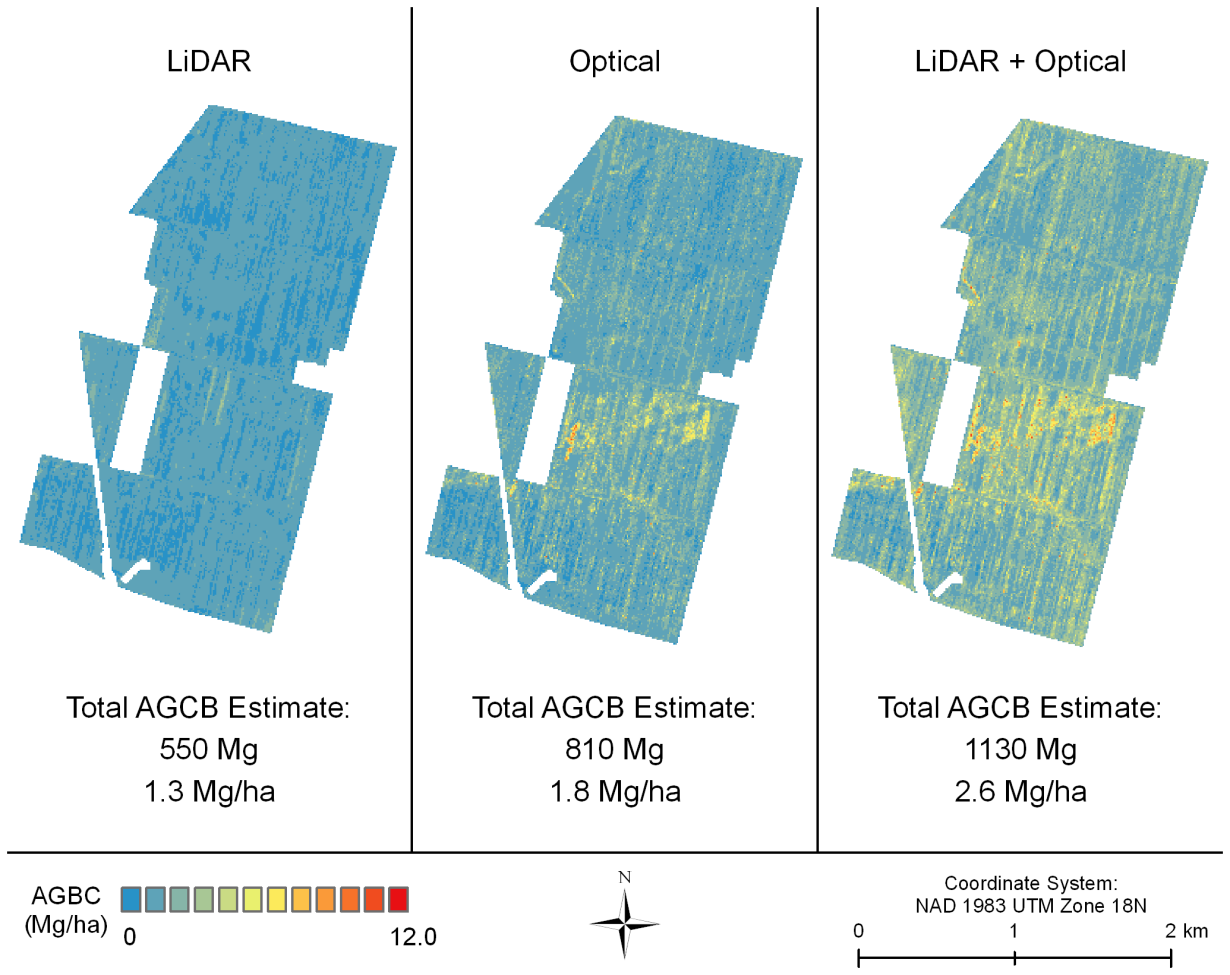
Maps of estimated AGCB created using the three remote sensing models. These maps were created by applying the biomass models to the respective remote sensing datasets which covered the entire study area. Note the differences in overall carbon biomass density and differences in the distribution of biomass at the study site as predicted by the three models.

The LiDAR model predicting AGCB was statistically significant (p<0.01); however, the fit of the model was relatively poor (adj-R^2^ = 0.18; RMSE = 0.17 Mg C/ha) (Table 5). The LiDAR model had difficulty accurately predicting the extreme high and low AGCB values (Figure 3 top panel). It tended to over-predict in areas of low observed biomass and under-predict in areas of high observed biomass. For instance, the maximum predicted AGCB value was only 3.51 Mg C/ha (Table 6), whereas the maximum observed AGCB value was 8.4 Mg C/ha (Table 4). As a result, the LiDAR model predicted a relatively homogeneous distribution of carbon at the study site (Figure 4, left panel). The LiDAR model predicted a total AGCB estimate of 550 Mg C (1.3 Mg C/ha).

**Table 5:** Regression equations for predicting AGCB (Mg C/ha) and total AGCB estimates.

Model	Equation	Estimated Total AGCB
LiDAR	{exp [3.25+0.60×log(LiDAR_Mean_Height)] - 1}/10	550 Mg C
Optical	{exp[3.78 - 1.40×log(NDVI_Mean) +4.80×log(NDVI_Max) - 1}/10	810 Mg C
LiDAR+Optical	{exp [4.33+0.28×log(Lidar_Mean_Height) - 1.05×log(NDVI_Mean) +	1130 Mg C
	3.96× (NDVI_Max)] - 1}/10	

**Table 6:** Descriptive statistics for model AGCB predictions over entire study area.

	Lidar	Optical	Lidar+Optical
n	44736	44736	44736
Mean (Mg C/ha)	1.23	1.80	2.53
Std. Dev. (Mg C/ha)	0.35	0.85	1.12
Min (Mg C/ha)	0.00	0.00	0.00
1st Q. (Mg C/ha)	1.03	1.29	1.81
Median (Mg C/ha)	1.21	1.65	2.35
3rd Q. (Mg C/ha)	1.41	2.11	3.02
Max (Mg C/ha)	3.51	10.69	11.25
Estimated Total AGCB (Mg C)	550	810	1130

The optical imagery model of AGCB was also statistically significant (p<0.001) (Table 5). Compared to the LiDAR model, the fit of the optical imagery model was somewhat better (adj-R^2^ = 0.34; RMSE = 0.14 Mg C/ha) and it had somewhat more success capturing the full range of AGCB values in the sample data (Figure 3, middle panel). The predicted range of carbon was between 0 Mg C/ha and 10.69 Mg C/ha (Table 6). While the optical imagery also tended to over-predict in areas of low biomass and under-predict in areas of high biomass, the amount of bias in the model was less than in the LiDAR model. As a result, the optical imagery model predicted a somewhat more heterogeneous distribution of biomass at the study site (Figure 4, middle panel). The optical imagery model predicted a total of 810 Mg C (1.8 Mg C/ha) for the study area.

Like the other two models, the combined LiDAR and optical imagery model was statistically significant (p<0.001) (Table 5). Including the variables from the two datasets led to a modest improvement in fit (adj-R^2^ = 0.37; RMSE = 0.14 Mg C/ha) compared to the optical imagery model. The combination model was also slightly more successful in predicting extreme high and low values, though bias was still evident (Figure 3, lower panel). The combination model predicted an even more heterogeneous distribution of biomass across the study site (Figure 4, right panel). The combined model predicted an AGCB range of 0 Mg C/ha to 11.25 Mg C/ha and a total of 1130 Mg C (2.6 Mg C/ha) (Table 6).

## Discussion

### Biomass Distribution

The most successful biomass models, the optical imagery and the combination models, both described significant heterogeneity in carbon biomass accumulation at the study site between 2004 and 2008. Ecologically, this finding is somewhat surprising given that all 750,000 trees were planted at the same time and were approximately the same size at the time of planting. In the predicted biomass maps for the optical imagery and the combination models (Figure 4, middle and right panels), two interesting patterns in biomass distribution are evident. First, there appear to be thin strips of relatively high biomass running approximately north - south throughout the study area. These correspond to the locations of old drainage ditches used for agriculture, which were filled in with top soil during the restoration process. We speculate that the higher productivity is the result of deeper soils and higher nutrient content where the former drainage ditches were filled. Second, the southern part of the old agriculture field appears to have areas of relatively high biomass. These are predominantly riverine areas which differ from non-riverine areas by being more frequently inundated with water and composed of different tree species. It is unclear, however, which factor – frequency of water saturation or species composition – is more responsible for the higher productivity in the southern part of the study site.

### Remote Sensing Model Performance

**Figure 5:**
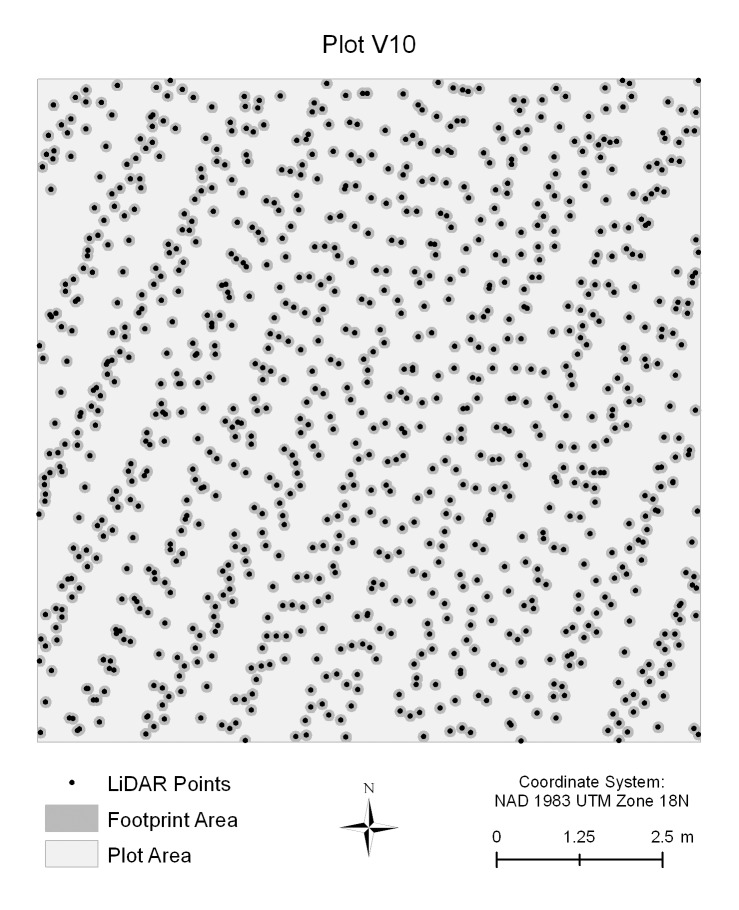
Map of vegetation plot V10 showing LiDAR point returns and pulse footprints. Among the 76 vegetation plots, V10 was the plot with the highest LiDAR pulse density (9.76/m^2^). The black dots represent the point returns. The dark gray areas around the black dots represent the LiDAR pulse footprints. Note the large areas (light gray) with no points which were effectively not sampled.

Our study represents the first attempt to use LiDAR to estimate above-ground carbon biomass in a recently planted restoration site. Compared with previous discrete-return LiDAR studies of mature forests, the LiDAR model in this study was much less successful, with an adj-R^2^ of just 0.18. The LiDAR model had particular difficulty estimating relatively high and relatively low biomass values. There are several factors that likely contributed to the LiDAR model’s relatively poor explanatory power compared to LiDAR models of mature forests. We speculate that the most significant factor is the relatively small stature of the trees at the study site. Due to their relatively small footprints, discrete-return LiDAR systems usually do not scan the whole ground surface. Instead, they merely sample the ground surface at a finite number of points. The ability of discrete-return LiDAR to detect and measure objects on the ground is a function of both the size of the objects as well as the sampling density. For the 76 vegetation plots in this study, the LiDAR pulse densities ranged from 5.4 to 9.8 pulses/m^2^, values that exceed those in previous, more successful discrete-return LiDAR biomass studies of mature forests [20], [21], [23]. This implies that the relatively poor performance of the LiDAR model was not simply due to a lower sampling density. Even with high pulse densities, however, the actual percentage of area scanned by a discrete-return LiDAR system can be quite low in absolute terms. In this study, the footprint for each pulse was approximately 16.25 cm in diameter, which means that only a small percentage (<20%) of each plot was effectively scanned (Figure 5). While even small effective sampling areas might be large enough to accurately estimate the heights of tall trees, they may not be large enough to accurately estimate the heights of small trees or even to detect them at all. We believe that this difficulty in detecting and measuring the heights of small trees contributed to relatively poor performance of the LiDAR model, in particular to the fact that the LiDAR model tended to underestimate the amount of carbon biomass in plots with a large number of small trees.

While we believe that the relatively short stature of the vegetation was the most significant cause of the relatively poor performance of the LiDAR model, other factors may have been influential as well. In 2008, the study site was covered with grasses and sedges in addition to planted trees. The presence of this herbaceous vegetation was reflected in the LiDAR data by a large percentage of non-ground returns within one meter of the ground surface. Returns from trees were extremely difficult to distinguish from low-elevation returns from herbaceous plants, a fact that helps to explain why the LiDAR model had difficulty distinguishing plots with high woody biomass from plots with low woody biomass but a lot of herbaceous plant material. The existence herbaceous vegetation is more likely to be a difficulty when modeling biomass in young forests than mature forests, and so this factor may help explain the relatively poor performance of the LiDAR model in this study compared to those in studies of mature forests. Another possible explanation is the fact that the LiDAR data was collected in mid-November, when leaf senescence was well underway. Loss of leaves probably contributed to the difficulty in detecting small trees, and it would help explain why the LiDAR model performed relatively poorly compared to previous studies that were conducted during a leave-on time period. However, this is not a sufficient explanation because at least one previous LiDAR study of tree height had successful results despite the fact that the discrete-return LiDAR data was collected during a leaf-off time period [22]. Finally, collecting reliable field biomass data for this study was complicated by the fact that most biomass allometric equations are based on diameter at breast height whereas only measurements of diameter at ground level were collected as part of the vegetation monitoring process. This necessitated the development of further allometric equations for predicting diameter at breast height from diameter at ground height. This extra step likely introduced some amount of error into the sample biomass data and may have contributed to the poor performance of all the models. Since this factor was specific to this study, it may also help explain why the LiDAR model in this study did not perform as well as those in previous discrete-return LiDAR biomass studies of mature forests.

In addition to suggesting that LiDAR-based models are unlikely to perform as well in areas with low-stature vegetation, our results also suggest that in these areas optical imagery models may outperform LiDAR-based models, counter to results that have been found in previous studies for mature forests. The main advantage LiDAR offers is the ability to retrieve information about the vertical structure of a forest with dense canopy cover. For recently planted or recolonized landscapes, where the tree-heights are low and the vertical structure of the vegetation is relatively homogeneous, LiDAR may have few advantages over optical imagery. However, the fact that the optical imagery was collected during a leave-on period whereas the LiDAR data was collected during a leaf-off period may also help explain why the optical imagery model performed better than the LiDAR model in this study.

### Conclusions and Recommendations

The results of this study suggest that while discrete-return LiDAR data has been used successfully to model biomass in mature forests, similar results should not necessarily be expected in areas with relatively short-stature vegetation. At the Timberlake study site, the absolute performance of the LiDAR model could be improved, however, by collecting the LiDAR data during a leave-on period and by developing methods for collecting more reliable field data (e.g. developing biomass allometric equations based on diameter-at-ground level instead of diameter-at-breast height). Nevertheless, the fact that smaller trees are more difficult to detect using discrete-return LiDAR suggests that, other things being equal, a drop-off in model performance, and perhaps a significant one, can be reasonably expected for areas with relatively short stature vegetation compared with areas of large trees.

In the context of a future carbon offset market, the expected difficulty of detecting small trees with discrete-return LiDAR should be considered when selecting a method for estimating carbon sequestration in an area that has been recently reforested or afforested. The results of this study suggest that optical imagery may prove to be the more reliable tool. However, given that all of the remote sensing models did a relatively poor job of capturing the observed variation in biomass (all adj-R^2^<0.4), it is unclear whether remote sensing methods are actually more reliable than the simpler method of scaling up from the sample data. We recommend more research comparing the abilities of both remote sensing methods and non-remote sensing methods for estimating carbon biomass in areas with relatively small trees. The difficulty of detecting small trees could be mitigated by increasing the pulse density of the collected discrete-return LiDAR data. Doing so, however, would make the LiDAR data acquisition more expensive due to the need for more over-flights of the aircraft. The problems associated with detecting small trees could perhaps also be mitigated by using full-waveform LiDAR instead of discrete-return LiDAR. Because full-waveform LiDAR pulses have larger footprints, they sample larger areas and thus are less likely to miss small trees. The downside of this approach, however, is that full-waveform LiDAR systems are not yet widely available for either scientific or commercial use. We also recommend that future research attempt to quantify the accuracies of biomass estimates as well as the economic tradeoffs. This information would be helpful for policy makers when choosing methods for estimating carbon sequestration methods in the context of a large-scale climate change mitigation program.

## References

[1] Zedler JB, Kercher S (2005) Wetland resources: Status, trends, ecosystem services, and restorability. Annu Rev Environ Resour 30: 39–74.

[2] Solomon SD, Qin D, Manning M, Chen Z, Marquis M, et al. (2007) Summary for Policymakers. In: Climate Change 2007: The Physical Science Basis. Contribution of Working Group I to the Fourth Assessment Report of the Intergovernmental Panel on Climate Change. Cambridge: Cambridge University Press. 18 p.

[3] Erwin KL (2009) Wetlands and global climate change: The role of wetland restoration in a changing world. Wetlands Ecol Manage 17: 71–84.

[4] Chatterjee R (2009) The Road to REDD. Environ Sci Technol 43: 557–560. 19244982 10.1021/es803353g

[5] Fletcher LS, Lena S, Kittredge D, Stevens T (2009) Forest Landowners’ Willingness to Sell Carbon Credits: A Pilot Study. Northern J Appl Forestry 26: 35–37.

[6] Hansen LT (2009) The viability of creating wetlands for the sale of carbon offsets. J Ag Res Econ 34: 350–365.

[7] Gibbs HK, Brown S, Niles JO, Foley JA (2007) Monitoring and estimating tropical forest carbon stocks: making REDD a reality. Environ Res Lett doi:10.1088/1748-9326/2/4/045023.

[8] Olander LP, Gibbs HK, Steininger M, Swenson JJ, Murray BC (2008) Reference scenarios for deforestation and forest degradation in support of REDD: a review of data and methods. Environ Res Lett doi:10.1088/1748-9326/3/2/025011.

[9] Asner GP (2009) Tropical forest carbon assessment: integrating satellite and airborne mapping approaches. Environ Res Lett doi:10.1088/1748-9326/4/3/034009.

[10] Lefsky MA, Cohen WB, Harding DJ, Parker GG, Acker SA, et al (2002) Lidar remote sensing of above-ground biomass in three biomes. Glob Ecol Biogeogr 11: 393–399.

[11] Evans JS, Hudak AT, Faux R, Smith AM (2009) Discrete Return Lidar in Natural Resources: Recommendations for Project Planning, Data Processing, and Deliverables. Remote Sens 1: 776–794.

[12] Lim K, Treitz P, Wulder M, St-Onge B, Flood M (2003) LiDAR remote sensing of forest structure. Prog Phys Geog 27: 88–106.

[13] Means JE, Acker SA, Harding DJ, Blair JB, Lefsky MA, et al (1999) Use of Large-Footprint Scanning Airborne Lidar to Estimate Forest Stand Characteristics in the Western Cascades of Oregon. Remote Sens Environ 67: 298–308.

[14] Drake JB, Dubayah RO, Clark DB, Knox RG, Blair JB, et al (2002) Estimation of tropical forest structural characteristics using large-footprint lidar. Remote Sens Environ 79: 305–319.

[15] Lefsky MA, Hudak AT, Cohen WB, Acker SA (2005) Geographic variability in lidar predictions of forest stand structure in the Pacific Northwest. Remote Sens Environ 95: 532–548.

[16] Gleason CJ, Im J (2010) A review of remote sensing of forest biomass and biofuel: options for small scale applications. GISRS 48: 141–170.

[17] Cohen WB, Spies TA (1992) Estimating structural attributes of Douglas-fir/western hemlock forest stands from Landsat and SPOT imagery. Remote Sens Environ 41: 1–17.

[18] Van Leeuwen M, Nieuwehnhuis M (2010) Retrieval of forest structural parameters using LiDAR remote sensing. Eur J Forest Res 129: 749–770.

[19] Smart LS, Swenson JJ, Christensen NL, Sexton JO (2012) Three-dimensional characterization of pine forest type and red-cockaded woodpecker habitat by small-footprint, discrete-return lidar. For Ecol Manage 281: 100–110.

[20] Hall SA, Burke IC, Box DO, Kaufmann MR, Stoker JM (2005) Estimating stand structure using discrete-return lidar: an example from low density, fire prone ponderosa pine forests. For Ecol Manage 208: 189–209.

[21] Popescu SC (2007) Estimating biomass of individual pine trees using airborne lidar. Biomass and Bioenergy 31: 646–655.

[22] Sexton JO, Bax T, Siqueira P, Swenson JJ, Hensley S (2009) A comparison of lidar, radar, and field measurements of canopy heights in pine and hardwood forests of southeastern North America. For Ecol Manage 257: 1136–1147.

[23] Gonzalez P, Asner GP, Battles JJ, Lefsky MA, Waring KM, et al (2010) Forest carbon densities and uncertainties from Lidar, QuickBird, and field measurements in California. Remote Sens Environ 114: 1561–1575.

[24] Ritchie JC, Humes KS, Weltz MA (1995) Laser altimeter measurements at Walnut Gulch watershed, Arizona. J Soil and Water Cons 50: 440–442.

[25] Næsset E, Bjerknes K (2001) Estimating tree heights and number of stems in young forest stands using airborne laser scanner data. Remote Sens Environ 78: 328–340.

[26] Streutker DR, Glenn NF (2006) LiDAR measurement of sagebrush steppe vegetation heights. Remote Sens Environ 102: 135–145.

[27] Wessels KJ, Mathieu R, Erasmus BF, Asner GP, Smit IP, et al (2011) Impact of communal land use and conservation on woody vegetation structure in the Lowveld savannas of South Africa. For Ecol Manage 261: 19–29.

[28] Carter LJ (1975) Agriculture: A New Frontier in Coastal North Carolina. Science 189: 271–275. 17813702 10.1126/science.189.4199.271

[29] Richardson CJ (1983) Pocosins: Vanishing wastelands or valuable wetlands? Bioscience 33: 626–633.

[30] Ardón M, Montanari S, Morse JL, Doyle MW, Bernhardt ES (2010) Phosphorous export from a restored wetland ecosystem in response to natural and experimental hydrologic fluctuations. J Geophys Res doi:10.1029/2009JG001169.

[31] Morse JL, Ardón M, Bernhardt ES (2012) Greenhouse gas fluxes in southeastern coastal plain wetlands under contrasting land uses. Ecol Appl 22: 264–280. 22471089 10.1890/11-0527.1

[32] Schlesinger WH (1991) Biogeochemistry, an Analysis of Global Change. New York: Academic Press. 458 p.

[33] Baltsavias EP (1999) Airborne laser scanning: basic relations and formulas. ISPRS J Photogramm Remote Sens 54: 199–214.

[34] Evans JS, Hudak AT (2007) A Multiscale Curvature Algorithm for Classifying Discrete Return LiDAR in Forested Environments. IEEE Trans Geosci Remote Sens 45: 1029–1038.

[35] Sellers PJ (1985) Canopy reflectance, photosynthesis and transpiration. Int J Remote Sens 6: 1335–1372.

[36] Jenkins JC, Chojnacky DC, Heath LS, Birdsay RA (2003) National-Scale Biomass Estimators for United States Tree Species. For Sci 49: 12–35.

[37] Clark AI, Phillips D, Frederick D (1985) Weight, volume, and physical properties of major hardwood species in the Gulf and Atlantic Coastal Plains. USDA For. Serv. Res. Pap. SE-250.

[38] Nelson L, Switzer G (1975) Estimating weights of loblolly pine trees and their components in natural stands and plantations in central Mississippi. Miss. Agric. And For. Exp. Sta. Tech. Bull. 73.

[39] Phillips D (1981) Predicted total-tree biomass of understory hardwoods. USDA For. Serv. Res. Pap. SE-223.

[40] Young HE, Ribe JH, Wainwright K (1980) Weight tables for tree and shrub species in Maine. Univ. of Maine Life Sci. and Agric. Exp. Sta., Maine Misc. Rep. 230.

